# Exercise-Induced Adult Cardiomyocyte Proliferation in Mammals

**DOI:** 10.3389/fphys.2021.729364

**Published:** 2021-08-30

**Authors:** Guo-liang Zhang, Ming-li Sun, Xin-an Zhang

**Affiliations:** College of Kinesiology, Shenyang Sport University, Shenyang, China

**Keywords:** exercise, cardiomyocyte, proliferation, molecular mechanism, cardiac repair

## Abstract

Loss of cardiomyocytes is a vital manifestation and predisposing factor of many cardiovascular diseases and will eventually lead to heart failure (HF). On the other hand, adult mammalian cardiomyocytes have a very limited regenerative capacity and cannot achieve self-repair of the myocardium after injury. Therefore, it is necessary to promote regeneration and repair of the myocardium through effective intervention means. Exercise plays an important role in the prevention and rehabilitation of cardiovascular diseases. Exercise can improve ischemia-reperfusion injury, reduce the size of the infarcted area, and improve the quality of life of patients. In addition, exercise has also been shown to be able to elevate the proliferative potential of adult cardiomyocytes and promote myocardial regeneration. Studies have shown that newly formed cardiomyocytes in adult mammalian hearts are mainly derived from pre-existing cardiomyocytes. By regulating various cytokines, transcription factors, and microRNAs (miRNAs), exercise can promote the dedifferentiation and proliferation of pre-existing cardiomyocytes to form new cardiomyocytes. Therefore, this paper focuses on the recent research progress of exercise-induced adult cardiomyocyte proliferation and explores its potential molecular mechanism.

## Introduction

Heart failure (HF) is an advanced stage of various cardiac diseases, placing a heavy burden on the human social health care system (Ziaeian and Fonarow, 2016). Myocardial injury for any reason, such as myocardial cell loss caused by myocardial infarction or apoptosis of cardiomyocytes in adverse cardiac remodeling, can cause myocardial structure and function changes. Eventually, it will lead to ventricular pumping and/or filling dysfunction, which triggers HF (Go et al., 2014; Wang et al., 2019). Therefore, early cardiac repair after the myocardial injury is an important method to avoid terminal HF. The regeneration of cardiomyocytes is a crucial factor in repairing cardiac function after injury. However, although studies have shown that the cardiomyocytes of adult mammals have a certain degree of proliferation ability (Bergmann et al., 2009), this ability is very limited. After the myocardial injury, the heart cannot repair spontaneously due to insufficient myocardial proliferative capacity (He and Zhou, 2017). In recent years, research on myocardial proliferation and regeneration has attracted more and more attention. Among them, using a variety of stem and progenitor cells to induce differentiation into cardiomyocytes has been considered a possible mechanism for cardiac repair after myocardial injury (Williams et al., 2013; Zhang et al., 2015). However, although this stem cell therapy has been gradually applied in clinical practice (Cahill et al., 2017; Sheng et al., 2019), there is still some controversy about its efficacy (Gyöngyösi et al., 2015). In addition, studies have demonstrated that pre-existing cardiomyocytes are the dominant source of cardiomyocyte replacement in normal mammalian myocardial homeostasis and after myocardial injury (Senyo et al., 2013). Therefore, how to promote the proliferation and differentiation of pre-existing cardiomyocytes to compensate for necrotic cardiomyocytes is the focus of current research. Previous studies have shown that exercise can induce endogenous cardiomyocyte proliferation of pre-existing cardiomyocytes (Boström et al., 2010). In addition, exercise is able to promote myocardial regeneration in the border zone of myocardial infarction (Vujic et al., 2018). However, there are few review articles on exercise and pre-existing cardiomyocytes, and there is a lack of exploration of their mechanisms. In this paper, we focus on the recent findings and possible mechanisms of exercise-promoted cardiomyocyte proliferation in order to provide a reference for further research and clinical application.

## Proliferative Potential of Adult Cardiomyocytes

Unlike certain lower vertebrates, which have a complete ability to regenerate the heart after injury, the adult mammalian cardiac tissue has been considered non-renewable for a long time (Becker et al., 1974; Zak, 1974; Poss et al., 2002). In comparison, it has been shown that the neonatal mammalian heart remains renewable after injury. It is achieved by partial dedifferentiation and complete division of pre-existing cardiomyocytes, and this ability is lost shortly after birth (Porrello et al., 2011; Zhu et al., 2018). It is believed that adult mammals cannot repair the necrotic myocardial tissue after myocardial injury but form extensive scar tissue (Uusimaa et al., 1997). However, with the research progress, a growing body of evidence shows that adult mammals still have heart regeneration capability to a certain extent, including humans (Hsieh et al., 2007^;^ Bergmann et al., 2009, 2015). By integrating carbon-14 produced by nuclear bomb tests during the cold war into cardiomyocyte DNA, the researchers found that the turnover rate of human cardiomyocytes gradually decreased from 1% per year at the age of 25–0.45% per year at the age of 75, and nearly 50% of cardiomyocytes were renewed during normal human life span (Bergmann et al., 2009). After myocardial infarction, cardiomyocyte proliferation can also be observed in adult hearts (Cai et al., 2016). Obviously, adult mammalian cardiomyocytes have an element of proliferative potential. However, this potential is not sufficient for mammalian hearts to repair themselves after injury (He and Zhou, 2017). Accordingly, research on the molecular mechanism of myocardial proliferation has attracted much attention in recent years. How to promote myocardial regeneration and cardiac repair in adult mammals has also become an intricate problem that has puzzled people for a long time.

## Cell Source of Regenerated Myocardium

To promote cardiomyocyte regeneration, we must first clarify the source of the regenerated cardiomyocytes. The regenerative capacity and cell source of cardiomyocytes have been studied for decades to pursue more practical therapeutic approaches. Nevertheless, limited by the research techniques and tools, there has been a lack of exploration of the proliferative capacity of mammalian mature cardiomyocytes. Only in recent years, with the revelation of the self-renewal capacity of adult cardiomyocytes and advances in research techniques, has there been a gradual focus on these pre-existing cardiomyocytes (Bergmann et al., 2009). A study by Senyo et al. (2013) that combined two different pulse-chase approaches – genetic fate-mapping with stable isotope labeling and multi-isotope imaging mass spectrometry showed that pre-existing cardiomyocytes were the primary source of cardiomyocyte turnover both in normal mammalian myocardial homeostasis and after myocardial injury. Subsequently, Liu et al. (2016) used an instant lineage tracing approach based on c-Kit antigen expression tracking, combined with long-term lineage tracing experiments, found that the vast majority of long-term labeled cardiomyocytes were pre-existing Kit-expressing cardiomyocytes, rather than cardiomyocytes formed by cardiac stem cells. Apparently, pre-existing cardiomyocytes play a significant role in the adult mammalian heart.

Moreover, it has also been shown that pre-existing cardiomyocytes expressing α heavy chains are the cell source for myocardial regeneration in neonatal mammals (Ali et al., 2014). In the adult heart, pre-existing mature cardiomyocytes achieve proliferation by re-entering the cell cycle and activating mitosis, which is also a potential pathway to promote myocardial regeneration. Studying the relevant targets and pathways is expected to provide a significant basis for improving clinical strategies to promote cardiac repair and regeneration.

## Mechanisms Underlying the Promotion of Adult Cardiomyocyte Proliferation by Exercise

**FIGURE 1:**
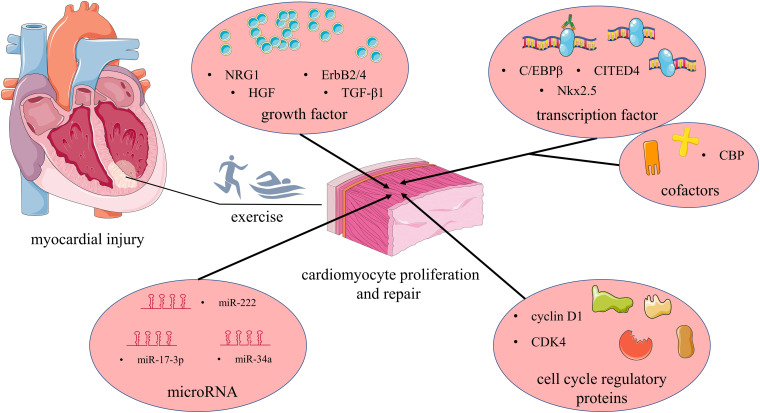
Mechanisms involved in exercise-induced cardiomyocyte proliferation and repair: after myocardial injury, exercise promotes cardiomyocyte proliferation, and repair by regulating growth factors, transcription factors, and transcriptional co-factors, cell cycle regulators, and miRNAs. NRG1, neuregulin 1; HGF, hepatocyte growth factor; TGF-β1, transforming growth factor-β1; CDK4, cyclin-dependent kinase 4; C/EBPβ, CCAAT enhancer-binding protein β; CITED4, CREB-binding protein [CBP]/p300-interacting transactivator with ED-rich carboxy-terminal domain-4; Nkx2.5, NK2 homeobox 5; CBP, cyclic-AMP-responsive element binding protein binding protein. Produced using Servier Medical Art (smart.servier.com).

As an important treatment, exercise has been shown to defense against cardiovascular disease and is included in treatment guidelines for cardiovascular disease by multiple institutions (Dickstein et al., 2008; Yancy et al., 2013; Lavie et al., 2015). Exercise can improve adverse cardiac remodeling, promote cardiac physiological hypertrophy, and improve cardiopulmonary function, thereby improving patient quality of life (Calvert et al., 2011; Donelli Da Silveira et al., 2020). In addition, there is accumulating evidence indicating that exercise can induce adult cardiomyocyte proliferation and promote the repair of the heart (Vujic et al., 2018). The above evidence provides hope for the treatment and rehabilitation of myocardial injury caused by a variety of diseases, but its mechanism of action is still far from clear. Therefore, as a valuable therapeutic strategy, it is necessary to explore the potential mechanism by which exercise promotes myocardial proliferation. Figure 1 provides an overview of molecular mechanisms in adult cardiomyocyte proliferation in response to physical exercise.

### Exercise Promotes Cardiomyocyte Proliferation by Regulating Growth Factors

Growth factors are a type of cytokine capable of stimulating cell growth activity. Many lines of evidence suggest important roles for some growth factors in promoting cardiac angiogenesis, extracellular matrix remodeling, cardiomyocyte proliferation, stem cell recruitment, as well as other cardioprotective effects (Rosenthal et al., 2006; Rebouças et al., 2016). Neuregulin 1 (NRG1), hepatocyte growth factor (HGF), and transforming growth factor-β1 (TGF-β1) are the main members of them. These three growth factors are closely related to the repair and regeneration of the myocardium. Besides, exercise has an apparent regulatory effect on them and may thus mediate the repair of the heart. First, NRG1/ErbB signaling is one of the mechanisms in exercise-induced cardiomyocyte proliferation. In a rat myocardial infarction model, Cai MX et al. found that the NRG1/ErbB signaling pathway was activated in the infarct border zone (Cai et al., 2016). At the same time, exercise further up-regulated the activity of the NRG1/ErbB pathway and enhanced cardiac function, indicating that exercise may improve myocardial infarction in rats by activating the NRG1/ErbB pathway. Further studies revealed that the number of cardiomyocytes with proliferative capacity was significantly increased after exercise intervention, and the ability to induce cardiomyocyte proliferation was decreased after blocking the NRG1/ErbB pathway (Cai et al., 2016). The above evidence suggests that NRG1/ErbB pathway is one of the critical pathways for exercise-induced cardiomyocyte proliferation, and exercise may improve myocardial infarction and promote cardiac repair by inducing cardiomyocyte proliferation through activation of this pathway.

Additionally, in a normal physiological rat model, aerobic exercise significantly increased the expression of HGF and TGF-β1 in myocardial tissue while increasing the number of cardiomyocytes with proliferative activity and enhancing the proliferative capacity of cardiomyocytes (Xing and Tian, 2018). The study indicates that aerobic exercise can promote cardiomyocyte proliferation in rats, and HGF and TGF-β1 may mediate this effect. It can be seen that some members of the growth factor family not only have positive effects in the protection and repair of the heart but may also act as regulators and play an important role in the process of exercise-induced adult cardiomyocyte proliferation. The precise mechanism still needs to be further explored for the previously mentioned growth factors and pathways with regulatory effects. While other growth factor family members, such as IGF1 and FGF10, are closely associated with cardiomyocyte proliferation and regeneration (Evans-Anderson et al., 2008; Rochais et al., 2014), whether they are involved in regulating exercise-induced myocardial proliferation is not clear and needs to be further explored.

### Exercise Promotes Cardiomyocyte Proliferation by Regulating Cell Cycle Regulators

Cell cycle activation is an essential condition for cardiomyocyte proliferation. The cell cycle is usually divided into four stages (cells in a quiescent state are called the G0 phase), which are G1 phase + S phase + G2 phase + M phase (Coffman, 2004). Cyclins, as well as their protein kinases (cyclin-dependent kinases, CDKs), are regulators with critical regulatory roles on the cell cycle (Hochegger et al., 2008). It has been shown that after birth, mammalian cardiomyocytes gradually lose their ability to proliferate and exit the cell cycle, and the cardiac growth pattern changes from proliferative to hypertrophic growth (Pasumarthi and Field, 2002; Porrello et al., 2011). Postnatal cell cycle exit is often accompanied by reduced expression of cyclins and CDKs, the inactivation of the cyclin-CDK complex, and increased expression of cyclin-dependent kinase inhibitors (CDKi) p21^cip1^ and p27^kip1^ (Poolman et al., 1998; Tane et al., 2014). Therefore, it is of great significance to study cell cycle regulators’ expression patterns to promote the re-entry of the cell cycle in cardiomyocytes and then promote cardiomyocyte proliferation. Mohamed TMA et al. showed that overexpression of cyclin D1 and CDK4, as well as two other regulators, could effectively induce cell division in post-mitotic mice, rat, and human cardiomyocytes; this demonstrates that discrete combinations of such a cell cycle regulator can effectively unlock the proliferative potential of cardiomyocytes that have exited the cell cycle (Mohamed et al., 2018). Exercise can promote cardiomyocyte proliferation and cardiac repair by intervening in the expression of the above cardiomyocyte cell cycle regulators. Studies have found that resistance exercise after myocardial infarction in rats can significantly up-regulate the protein expression of FSTL1 and its receptor DIP2A, effectively activate the PI3K/Akt pathway and significantly increase the protein expression of CyclinD1 and CDK4 (Tian et al., 2018); At the same time, resistance exercise after infarction increased the number of cardiomyocyte with proliferative activity, significantly increased the percentage of cardiomyocyte proliferation, and significantly improved cardiac function (Tian et al., 2018); the above evidence suggests that resistance exercise may play a cardioprotective role by activating the FSTL1-DIP2A-Akt-mTOR signaling pathway and up-regulating the expression of CyclinD1 and CDK4, thereby initiating the cell proliferation cycle and participating in cardiomyocyte proliferation. In addition, although aerobic exercise can also reduce the expression of negatively regulated genes of the cell cycle in rat cardiomyocytes, the correlation between this regulatory effect and cardiomyocyte proliferation has not been confirmed (Mota et al., 2020). On the other hand, at present, the activation of some cell cycle activity or DNA synthesis markers does not fully prove the actual proliferation and generation of cardiomyocytes, and we still lack direct evidence for this; however, this does not prevent them from being widely used as evaluation indicators of cardiomyocyte proliferation (Boström et al., 2010; Huang et al., 2018; Mohamed et al., 2018).

### Exercise Promotes Cardiomyocyte Proliferation by Regulating Transcription Factors and Co-factors

Transcription factors are a class of protein factors whose primary function is to assist eukaryotic transcription. Some of these members, such as Nkx2.5, Tbx5, and Gata4, play an important role in cardiac development, cardiomyocyte differentiation, and some cardiac diseases (Akazawa and Komuro, 2005; Paige et al., 2015; Werner et al., 2019). Therefore, the role of transcription factors in the regulation of cardiomyocyte proliferation has also been extensively studied. Boström et al. (2010) found that endurance exercise could not only induce physiological cardiac hypertrophy but also significantly increase the proliferation rate of cardiomyocytes in a mice model; also, decreased expression of CCAAT enhancer-binding protein β (C/EBPβ) can be observed in the hearts of these exercised mice; interestingly, *in vitro* experiments using primary rat cardiomyocytes also found that reducing the expression of C/EBPβ can lead to an increase in the number of cardiomyocytes accompanied by an enhancement of cell proliferation activity; this suggests that C/EBPβ is one of the key transcription factors involved in regulating exercise-induced cardiomyocyte proliferation. In addition, the transcription factor CBP/p300-interacting transactivator with ED-rich carboxy-terminal domain 4 (CITED4) showed a robust increase in exercised hearts; *in vitro* studies have shown that forced expression of CITED4 increases the number of cardiomyocytes while increasing the percentage of cardiomyocytes with proliferative activity; in contrast, blocking CITED4 reduced cardiomyocyte number and cardiomyocyte proliferative activity, and this was also confirmed *in vivo* in mice after exercise (Boström et al., 2010; Bei et al., 2017). Furthermore, CITED4 has a close link with C/EBPβ; with the gain or loss of function of C/EBPβ, the expression level of CITED4 was significantly changed, indicating that CITED4 may function as a downstream factor of C/EBPβ; however, CITED4 knockout completely abolished the cell proliferation effect caused by C/EBPβ reduction, indicating that C/EBPβ may mediate its anti-proliferative effect at least in part through the expression of CITED4 (Boström et al., 2010). Taken together, we have reason to believe that exercise can induce cardiomyocyte proliferation by inhibiting C/EBPβ and up-regulating CITED4 expression and may therefore play an important role in resistance to adverse cardiac remodeling and subsequent HF. The study of Naderi et al. (2019) in adult rat heart further showed that exercise training after myocardial infarction could promote the repair of infarcted myocardium; in addition to C/EBPβ and CITED4, factors such as GATA4, Nkx2.5, Tbx5, and Mef-2c may also be involved in exercise-induced cardiomyocyte proliferation. However, whether there are targets downstream of C/EBPβ, whether CITED4 independently regulates cardiomyocyte proliferation, and the exact role of factors such as GATA4 still need further discussion.

The cyclic-AMP-responsive element binding protein binding protein (CBP), a transcriptional coactivator, is a crucial regulator of RNA polymerase II-mediated transcription (Teo and Kahn, 2010). CBP can bind to a variety of transcription factors and regulate gene transcription and protein expression. Studies have shown that aerobic exercise can significantly activate the proliferation ability of cardiomyocytes, accompanied by an increase in the mRNA and protein expression levels of CBP; inhibition of the expression level of CBP can inhibit the proliferation ability of cardiomyocytes, indicating that CBP plays an essential regulatory role in the process of aerobic exercise promoting cardiomyocyte proliferation (Hu et al., 2016).

### Exercise Promotes Cardiomyocyte Proliferation by Regulating MicroRNAs

MicroRNAs (miRNAs) are a class of non-coding single-stranded RNA molecules of approximately 22 nucleotides in length encoded by endogenous genes, and miRNAs are involved in post-transcriptional gene expression regulation by binding to complementary sequences in the coding or 3′ untranslated regions of mRNAs (Huang et al., 2011). Recent studies have shown that miRNAs play an important role in the physiological and pathological mechanisms of many cardiovascular diseases and regulate processes such as cardiomyocyte proliferation, apoptosis, and metabolism (Dawson et al., 2013; Chen et al., 2014; Wang and Martin, 2014; Ultimo et al., 2018). Among them, some miRNAs have been found to contribute to promoting cardiac repair after cardiac injury. Shi et al. (2017) found in a mouse model that miR-17-3p was able to promote cardiomyocyte proliferation and hypertrophy; its regulatory mechanisms included direct inhibition of TIMP-3 and indirect inhibition of PTEN; mice treated with miR-17-3p protein could prevent adverse cardiac remodeling after myocardial ischemia, implying that miR-17-3p may become a new target to promote functional repair after myocardial ischemia/reperfusion. Meanwhile, miR-17-3p was also shown to be a key regulator of exercise-induced cardiomyocyte growth and proliferation in mice. Exercise training was able to increase the level of miR-17-3p in mouse hearts significantly, and inhibition of miR-17-3p *in vivo* attenuated exercise-induced cardiac growth, including cardiomyocyte hypertrophy and expression of cardiomyocyte proliferation markers (Shi et al., 2017). Similarly, miR-222 also promotes cardiomyocyte proliferation and fight against adverse cardiac remodeling and dysfunction after ischemic injury, and these effects are achieved by directly regulating downstream targets p27, Hipk1, and Hmbox1 (Liu et al., 2015). In addition, miR-222 was significantly up-regulated in mouse hearts after swimming training and spontaneous wheel exercise. The inhibition of miR-222 completely blocked exercise-induced mice cardiac growth while reducing markers of cardiomyocyte proliferation (Liu et al., 2015; Vujic et al., 2018). These studies implicate that miR-222 is necessary for exercise-induced cardiomyocyte growth and proliferation in the adult mammalian heart.

Furthermore, mature miR-34a was shown to be regulated by ADAR2, which is involved in exercise-induced cardiomyocyte proliferation (Wu et al., 2021). ADAR2 is an RNA-editing enzyme, which belongs to the Adenosine deaminases acting on RNA (ADAR) family of enzymes that catalyzes the conversion of adenosine to inosine (A–I) in pre-mRNA. Wu et al. (2021) found that swimming training was able to up-regulate ADAR2 expression in the heart in mice and rat models; *in vitro* studies have demonstrated that overexpression of ADAR2 can induce proliferation of primary neonatal rats cardiomyocytes, indicating that exercise may promote cardiomyocyte proliferation by regulating ADAR2. Additionally, the expression of miR-34a was decreased in the exercised hearts; meanwhile, miR-34a is negatively regulated by ADAR2, with the overexpression and knockdown of ADAR2, the expression level of miR-34a was correspondingly changed; on the other hand, pro-proliferation effects of ADAR2 were mediated by miR-34a inhibition, and overexpression of miR-34a could block the protective effect of ADAR2 (Wu et al., 2021). The above evidence indicates that ADAR2/miR-34a axis plays an essential role in exercise-induced cardiomyocyte proliferation. In addition to the above miRNAs, miR-590, and miR-199a have also been shown to promote the proliferation and regeneration of adult cardiomyocytes, but their association with exercise remain to be examined. Table 1 gives an overview of factors related to exercise-induced cardiomyocyte proliferation.

**TABLE 1:** Factors related to exercise-induced cardiomyocyte proliferation.

Classification	Factor	Exercise modality	Animal model	Modulating effects of exercise	References
Growth factors	NRG1, ErbB, and PI3K	Treadmill running	Rats	NRG1 expression ↑	Cai et al., 2016
				Activity of ErbB2, ErbB4, and PI3K/Akt pathway ↑	
	HGF and TGF-β1	Treadmill running	Rats	HGF expression ↑	Xing and Tian, 2018
				TGF-β1 expression ↑	
Cell cycle regulators	Cyclin D1 and CDK4	Resistance exercise	Rats	Cyclin D1 expression ↑	Tian et al., 2018
				CDK4 expression ↑	
Transcription factors and co-factors	C/EBPβ and CITED4	Swimming exercise	Mice	C/EBPβ expression ↓	Boström et al., 2010
				CITED4 expression ↑	
	C/EBPβ, CITED4, GATA4, Nkx2.5, Tbx5, Mef-2c	Treadmill running, high-intensity interval training	Rats	C/EBPβ expression ↓	Naderi et al., 2019
				Expression of CITED4, GATA4, Nkx2.5, Tbx5, and Mef-2c ↑	
	CBP	Swimming exercise	Mice	CBP expression ↑	Hu et al., 2016
MicroRNA	miR-17-3p	Swimming exercise	Mice	miR-17-3p expression ↑	Shi et al., 2017
		Voluntary wheel-running			
	miR-222	Swimming exercise	Mice	miR-222 expression ↑	Liu et al., 2015; Vujic et al., 2018
		Voluntary wheel-running			
	miR-34a	Swimming exercise	Mice	miR-34a expression ↓	Wu et al., 2021

## Application Value of Exercise in Promoting the Proliferation of Adult Cardiomyocytes

As an important part of cardiac rehabilitation, the cardiovascular protective effect of exercise has been generally recognized. As a non-invasive treatment, exercise has a vital role in preventing cardiovascular risk factors. Exercise can not only lower blood pressure, lower blood glucose, improve insulin resistance and lipid metabolism abnormality (Weiss et al., 2016; Duvivier et al., 2017), but also defend against cardiac diseases such as cardiac ischemia-reperfusion injury, coronary heart disease, and HF; besides, exercise can significantly improve the quality of life of patients (Calvert et al., 2011; Stewart et al., 2017; Donelli Da Silveira et al., 2020). Study has shown that exercise-induced cardiac physiological growth is not affected when exercise-induced cardiomyocyte proliferation is attenuated, while the effect of exercise on reducing cardiac ischemia-reperfusion injury disappears (Bei et al., 2017). This evidence demonstrates that cardiomyocyte proliferation is unnecessary for exercise-induced cardiac physiological growth, but it is necessary for exercise to prevent ischemia-reperfusion injury (Bei et al., 2017). In addition, Vujic et al. (2018) demonstrated in a mice model that exercise training after myocardial infarction can induce a strong cardiomyocyte regenerative response at the border of the infarcted area. In a rat model of myocardial infarction, interval training has also been shown to induce cardiomyocyte proliferation and significantly reduce infarct size (Shi et al., 2016). However, though many animal experiments show that exercise can promote the repair of infarcted myocardium by inducing endogenous cardiomyocyte proliferation (Cai et al., 2016; Tian et al., 2018; Naderi et al., 2019), it remains unknown whether the same effect can be achieved in humans due to methodological difficulties in assessing cardiomyocyte regeneration and turnover in humans. However, as shown above, with the continuous progress of research technology, many advanced methods, such as multi-isotope imaging mass spectrometry, genetic fate mapping, mosaic analysis with double markers, and lineage tracing technology, have been applied to the study of cardiomyocyte proliferation. Therefore, with the birth and application of more advanced technologies and experimental designs, it is reasonable to believe that the effect of exercise on human adult cardiomyocyte proliferation and cardiac repair will eventually be revealed.

## Summary and Prospect

Unlike traditional cognition, adult mammalian cardiomyocytes have some proliferative potential. This potential is achieved mainly by the proliferation of pre-existing cardiomyocytes. In recent years, pre-existing cardiomyocytes have gradually entered the field of view and attracted greater attention. Exercise can significantly promote the proliferation of pre-existing cardiomyocytes by regulating growth factors, transcription factors and co-factors, cell cycle regulators, and miRNAs. By inducing cardiomyocyte proliferation, exercise has also been shown to ameliorate ischemia-reperfusion injury and reduce the size of myocardial infarction, thereby promoting cardiac repair. However, the cardiac repair effect of exercise still lacks to be proved by relevant clinical experiments, and the detailed mechanism is far from clear. In addition, there are still many drawbacks to be solved in this field, which can be roughly summarized into the following four aspects: First, there are few existing studies on the mechanisms of exercise and pre-existing cardiomyocytes, and most of them are indirect mechanism studies, there is a lack of direct mechanism studies. Second, the current study lacks attention to the uniqueness of exercise; that is, no study has demonstrated which mechanisms are unique to exercise-induced adult cardiomyocyte proliferation. Third, although there is evidence that exercise can promote cardiomyocyte proliferation under both normal physiological and pathological injury conditions, comparative studies are lacking to demonstrate whether there are differences in the mechanisms of exercise-induced cardiomyocyte proliferation under physiological and pathological conditions. Finally, an exercise program is usually composed of four parts: the modality, intensity, frequency, and duration of exercise. Different exercise protocols may cause different effects on cardiac regeneration. However, we still lack relevant systematic studies on how different exercise protocols affect cardiac regeneration. Therefore, the joint efforts of more relevant researchers and experts are needed to perform systematic experimental design and experimental analysis, explore reliable regulatory mechanisms and establish an effective evaluation system, so that exercise can effectively benefit patients with cardiovascular diseases.

## Author Contributions

X-AZ and M-LS: conceptualization, project administration, and funding acquisition. G-LZ, X-AZ, and M-LS: writing – review and editing. All authors contributed to the article and approved the submitted version.

## Conflict of Interest

The authors declare that the research was conducted in the absence of any commercial or financial relationships that could be construed as a potential conflict of interest.

## Publisher’s Note

All claims expressed in this article are solely those of the authors and do not necessarily represent those of their affiliated organizations, or those of the publisher, the editors and the reviewers. Any product that may be evaluated in this article, or claim that may be made by its manufacturer, is not guaranteed or endorsed by the publisher.
